# Activated Eosinophils in Association with Enteric Nerves in Inflammatory Bowel Disease

**DOI:** 10.1371/journal.pone.0064216

**Published:** 2013-05-22

**Authors:** Claire M. Smyth, Nadim Akasheh, Sara Woods, Elaine Kay, Ross K. Morgan, Margaret A. Thornton, Anthony O’Grady, Robert Cummins, Orla Sheils, Peter Smyth, Gerald J. Gleich, Frank M. Murray, Richard W. Costello

**Affiliations:** 1 Departments of Medicine, Royal College of Surgeons in Ireland, Beaumont Hospital, Dublin, Ireland; 2 Department of Pathology, Royal College of Surgeons in Ireland, Beaumont Hospital, Dublin, Ireland; 3 Department of Dermatology, School of Medicine, Salt Lake City, Utah, United States of America; University of Montreal, Canada

## Abstract

Enteric neural dysfunction leads to increased mucous production and dysmotility in inflammatory bowel disease (IBD). Prior studies have shown that tissue eosinophilia is related to disease activity. We hypothesized that interactions between eosinophils and nerves contribute to neural dysfunction in IBD. Tissue from patients with intractable IBD, endoscopic biopsies from patients with steroid responsive IBD, both when active and quiescent, and control tissue were studied. Immunohistochemical studies showed that eosinophils localize to nerves in the mucosal layer of patients with Crohn’s disease (CD) (p<0.001) and ulcerative colitis (UC), (p<0.01). Eosinophils localized to substance P and choline acetyltransferase (ChAT) immunostained nerves. Real time PCR of laser capture micro-dissected enteric ganglia demonstrated Intercellular Adhesion Molecule 1 (ICAM-1) mRNA was increased 7-fold in UC (n = 4), (p = 0.03), and 10-fold in CD (n = 3), (p = 0.05). Compared with controls, eotaxin-3 (CCL-26) mRNA was increased 9-fold in UC (p = 0.04) and 15-fold in CD (p = 0.06). Eosinophil numbers correlated with disease activity, while deposition of major basic protein (MBP) and eosinophil Transforming Growth Factor β -1 (TGFβ-1) expression were seen in therapeutically responsive disease. These data indicate a significant localization of eosinophils to nerves in IBD, mediated through neurally expressed ICAM-1 and eotaxin-3. This cell/neural interaction may influence the function of nerves and contribute to symptoms in IBD.

## Introduction

The inflammatory bowel diseases (IBD), ulcerative colitis (UC) and Crohn’s disease (CD) are relatively common clinical conditions which are characterized by the symptoms of bloody diarrhea and excessive mucous production. Histologically, IBD is associated with a marked inflammatory cell infiltrate and varying degrees of mucosal ulceration. Despite increased information on the clinical course and significant advances in the treatment of these diseases, there is still a lack of information on the mechanisms of these symptoms.

The bowel is innervated with an extensive neural network and this innervation is important, not just in normal physiological function but also as part of the host’s response to enteric injury [1], [2]. Increased enteric neural activity leads to enhanced smooth muscle contraction and mucous production, altered local blood flow, recruitment of inflammatory cells and the sensation of pain [3]–[5]. Many of the symptoms of IBD, such as diarrhea and mucous production may be due to increased neural activity. In addition to the altered neural activity seen in IBD, the mucosa is infiltrated with a variety of inflammatory cells including eosinophils [6]–[10]. A considerable body of evidence supports both pathological and possibly beneficial roles for eosinophils in IBD [11]. Reduced colonic eosinophilia in eotaxin knockout mice has been shown to attenuate experimental colitis [12]. Eosinophils may interfere with normal cellular function by the release of cationic proteins such as major basic protein (MBP) [13] which, in high concentrations, is toxic to cells [14], [15]. Animal studies have suggested a role for eosinophil cationic proteins in the pathogenesis of IBD [16]–[18]. Increased levels of eosinophil degranulation products have also been detected in the faeces and intestinal lavage fluid of patients with inflammatory bowel disease [19], [20]. Serological Eosinophil Cationic Protein (ECP) and Eosinophil Protein X levels, however, have not been shown to reflect the degree of eosinophilic colonic inflammation [21]. On the other hand eosinophils may also contribute to the host’s response to infection, through the antibacterial effects of the eosinophil granular proteins [22], [23] or cause remodelling through the release of either neurotrophins [24] or transforming growth factor-β-1 (TGFβ-1) [25]. We and others have previously reported that there are considerable interactions between eosinophils and nerve cells in a number of pathological conditions [26]–[31]. In particular, in vivo, in the airways of antigen challenged animals and humans with asthma we have shown that eosinophils influence nerve function, through the release of major basic protein (MBP) onto muscarinic M_2_ receptors [32]–[34]. Inhibition of these receptors by MBP is associated with increased vagally-mediated smooth muscle contraction [35]. Other investigators have shown that eosinophils can influence the release of neuropeptides such as substance P [36] and calcitonin gene-related peptide (CGRP) [37]. Thus, we hypothesized that eosinophil interactions with nerves may be a mechanism whereby eosinophils lead to the altered neural function in IBD. Furthermore, we hypothesized that if there was an association of eosinophils with nerves that there may be a specific mechanism of localization through neural expression of adhesion molecules and chemoattractants. We addressed this hypothesis by defining the nerve cell and eosinophil interactions in patients with acute exacerbations, quiescent disease and a separate group who had therapeutically resistant IBD.

## Materials and Methods

### Ethics Statement

Ethical approval for this study was obtained from the Beaumont Hospital Ethics (Medical Research) committee. Written consent was obtained in all cases.

### Materials

The source, concentration, antigen retrieval methods and detection systems for antibodies used in this study are shown in Table 1. The affinity-purified polyclonal rabbit antibody to human eosinophil MBP was used to identify eosinophils [38]. Trilogy/Declere solution was purchased from Cell Marque, Hot Springs, Arkansas, USA. Pronase was purchased from DAKO (Copenahagan, Denmark, Cat. No.S2013). The Vectastain universal ABC-AP kit, Vectastain ABC kit and Chromogens were obtained from Vector Laboratories. Flourescein Avidin D, Texas Red Avidin D and Avidin/Biotin blocking kit were all purchased from Vector Laboratories (Burlingame, CA, USA). 4′, 6-Diamino-2-phenylindole dihydrochloride (DAPI) was purchased from Chemicon International, CA, USA. Laser capture microdissection was performed using a Pix-Cell II instrument from Arcturus Engineering (Mountain View, CA). RNA isolation was carried out using the Purescript RNA Isolation Kit purchased from Gentra Systems. Quantitative PCR was performed using the Lightcycler purchased from Roche Molecular Biochemicals, Lewes, UK. First Strand cDNA Synthesis Kit and double-stranded DNA binding dye SYBR Green 1 (Fast Start DNA Masters SYBR Green 1) were also purchased from Roche Molecular Biochemicals, Lewes, UK.

**Table 1:** Details of antibodies used in the study.

ANTIBODY	CONC.	ANTIGEN RETRIEVAL	INCUBATION	DETECTION	SOURCE	Ab TYPE
**MBP^^^**	**1∶100**	**Pronase**	**60 min**	**Vec. Red** *		**Polyclonal**
**S100**	**1∶2000**		**30 min**	**DAB**	**DAKO**	**Polyclonal**
MBP	1∶100		Overnight	Vec. Red		Polyclonal
SubP^♦^	1∶150	Trilogy	60 min	DAB	Zymed	Polyclonal
**MBP**	**1∶100**		**Overnight**	**Vec. Red**		**Polyclonal**
**nNOS^♠^**	**1∶400**	**Trilogy**	**60 min**	**DAB**	**Upstate**	**Polyclonal**
MBP	1∶100		60 min	Vec. Red		Polyclonal
ChAT^•^	1∶25	Trilogy	60 min	DAB	Chemicon	IgG_1_
**TGFβ-1°**	**1∶40**	**Pronase**	**Overnight**	**Vec. Red**	**R&D**	**IgG_1_**
**S100**	**1∶2000**		**30 min**	**DAB**	**DAKO**	**Polyclonal**
TGFβ-1	1∶40	Pronase	Overnight	FAD	R&D	IgG_1_
MBP	1∶100		60 min	TRAD		Polyclonal

The antibodies used for the immunostaining in this study, their source, type, concentration, method of antigen retrieval and detection are shown.

* Vector Red ∧Major basic protein ♦Substance ♠P Neuronal nitric oxide synthase •Choline acetyltransferase °Transforming growth factor-beta-1 DAB – diamino benzidine FAD – Fluorescein Avidin D TRAD – Texas Red Avidin D.

### Study Groups

Two different groups of patients with IBD were investigated. Full thickness sections of bowel wall were obtained from subjects undergoing large bowel resection for disease which was refractory to medical treatment. The subject characteristics are shown in **Table 2**. The diagnostic criteria of IBD were based on standard clinical, radiological, endoscopic and histological findings [39]. Control tissue was taken from the distal (normal) end of resected colorectal tumors (n = 8) (**Table 2**).

**Table 2:** Characteristics of patients with refractory IBD and controls.

DIAGNOSIS	CLASSIFICATION	GENDER/AGE	DISEASE DURATION	MEDICATION
**Crohn’s Disease**	Stricturing and fistulating	Female/30	6 years	AZA
	Inflammatory	Male/17	Acute presentation	None
	Inflammatory stricture	Male/26	3 years	M, S
	Stricturing	Female/52	6 years	M
	Stricturing	Female/45	17 years	S
	Stricturing and fistulating	Male/28	11 years	M, S
	Stricturing	Female/33	7 years	Sz, S
	Stricturing and fistulating	Male/27	8 years	ED
**Ulcerative Colitis**	Distal	Female/39	18 months	O, S po/iv/pr
	Pancolitis	Male/25	4 weeks	M, S po/iv/pr, Cy
	Pancolitis	Female/35	2 years	O, S po/iv
	Pancolitis	Male/33	3 years	M po/pr, S po/iv
	Pancolitis	Male/39	3 weeks	M, S po/iv/pr
	Proctitis	Female/34	8 years	Sz, S po/iv
	Pancolitis*	Female/39	2 weeks	C, S po/iv
**Controls**	Caecal Ca	Male/64		Eso
	Rectal Ca	Female/76		El
	Ascending CRC	Male/46		None
	Rectal Ca	Female/76		None
	Ascending CRC	Male/73		NSA, Fe, B12
	Rectal Ca	Female/41		ACEI
	Ascending CRC	Male/74		PPI, Fe
	Descending CRC	Male/64		S, PPI

This table outlines the demographics of the patients with refractory IBD and control patients used in this study.

* Multiple Sclerosis for years; CRC – colorectal carcinoma; po – per oral; pr – per rectum; iv – intravenously; AZA – Azathioprine; M – Mesalazine; S – Steroids; Sz – Salazopyrine; ED – Elemental Diet; O – Olsalazine; C – Cyclophosphamide; Cy – Cyclosporin; Eso – Esomeprazole; El – Eltroxin; PPI – proton pump inhibitor; Fe – iron supplements; NSA – nuseals aspirin; ACEI – angiotensin converting enzyme inhibitor.

A second group included 11 patients with therapeutically responsive ulcerative colitis. Mucosal biopsies were obtained from these subjects when the disease was clinically symptomatic and again when the disease was quiescent (**Table S1**). Active disease was diagnosed according to standard diagnostic criteria and activity indices [40], [41]. The patients with active disease were treated according to standard practice [42], [43]. Biopsies were similarly obtained when the subjects were clinically, endoscopically and histologically quiescent.

### Double Immunostaining for Eosinophil MBP and Nerve Subspecies

The tissue specimens were immediately fixed in 4% formalin and subsequently embedded in paraffin blocks. The blocks were cut into 4 µm thick sections and mounted on adhesive slides. The tissues were de-waxed by immersion in xylene, re-hydrated in alcohol and washed in distilled water. In the case of the nerve subspecies, namely substance P (Sub P), neuronal nitric oxide synthase (nNOS) and choline acetyltransferase (ChAT), tissues were prepared using the Trilogy/Declere solution method, as described by the manufacturer. Trilogy/Declere pressurizes the tissue in a pressure cooker for 15 minutes, and combines the three steps of dewaxing, rehydration and unmasking of antigenic sites. Non-specific peroxidase activity was then blocked by immersing the slides in 3% hydrogen peroxide, after two washes in Tris-buffered-saline (TBS) the tissue was then incubated with 10% normal horse serum for 10 minutes followed by the application of the polyclonal rabbit anti-human MBP antibody. After incubation with the antibody, the tissue was then washed twice in TBS, incubated for 20 minutes with a biotinylated horse secondary antibody, washed twice in TBS and incubated for a further 20 minutes with an avidin biotinylated alkaline phosphatase complex. Detection was with the Vector Red substrate. This produced a red coloration within eosinophils and also allowed identification of extra-cellular MBP. After eosinophil MBP immunostaining, the second primary antibody was applied to the tissues, as outlined in **Table 1**. Following incubation with a biotinylated universal secondary antibody and universal avidin biotinylated complex, detection was with incubation with diamino benzidine (DAB) which resulted in a brown color of the nerves. Control methods consisted of omission of the primary antibody. Individual parallel IgG controls were also used in addition to primary antibody omission during the preliminary testing of these antibodies. Analysis revealed no difference between these two methods of control. Hence, for the remainder of the study negative controls consisted of sections with primary antibody omitted. The tissues were counterstained with dilute haematoxylin, 1% acid alcohol and lithium carbonate.

### Laser Capture Micro-dissection (LCM)

Tissue sections from patients with refractory IBD and controls were used for this part of the study (CD n = 3, UC n = 4, Controls n = 5). The sections for laser capture micro-dissection were prepared from formalin fixed paraffin-embedded sections of resected colon. Sections were cut to a 6 µm thickness and placed on a non-adhesive slide. The sections were air-dried and subsequently incubated overnight at 55°C. The sections were then immunostained using an anti-S100 antibody as a general nerve marker as described above. On average four full-thickness sections from each patient were used for LCM. From each section, between 20 and 40 ganglia were dissected. Initially, non-specific tissue and dust was cleared from the section using a Capsure Cleanup Pad (Arcturus). Laser capture micro-dissection was performed using a Pix-Cell II system. Nerve ganglia stained with anti-S100 were captured using either the 7.5 µm or the 15 µm laser setting depending on the size of the nerve to be captured. The laser was set to a pulse of 100 mW. Images were collected using the PixCell II Image Archiving Workstation.

### RNA Isolation

RNA isolation was carried out using the Purescript RNA Isolation Kit according to the manufacturer’s recommendations. Capture disks with the isolated neuronal tissue were attached to 0.5 ml ependorfs containing 300 µl of cell lysis solution (Gentra Systems) and 1.5 µl of proteinase K. These were placed in a 55°C oven overnight, rotating continuously. The following day, 100 µl of Protein-DNA Precipitation Solution (Gentra Systems) was added to the cell lysate, this was subjected to centrifugation and the supernatant was collected, removed and placed in a new ependorf to which 300 µl of ice-cold isopropanol plus 0.5 µl of glycogen were added. The tubes were inverted 50 times and placed in a −20°C freezer for 30 minutes. The samples were then centrifuged at 15,000 rpm for a further 3 minutes. The supernatant was discarded and 300 µl of 70% ethanol was added to the tubes that were inverted gently 10 times and further centrifuged at 15,000 rpm for one minute. The supernatant was discarded and samples were blot dried and then allowed to further air dry. Once fully dry, the pellet was re-suspended in 25 µl of RNA Hydration Solution (Gentra Systems) and stored at −80°C, until further analysis. Samples from each group of diseased subjects and the controls were pooled for quantitative PCR.

### Real Time Quantitative PCR

For quantitative Lightcycler PCR, 1 µg of total RNA was reverse transcribed into cDNA with an oligo(dT)_15_ primer by means of the First Strand cDNA Synthesis Kit. Amplification of cDNA was carried out by quantitative PCR in a Lightcycler in the presence of the double-stranded DNA binding dye SYBR Green 1. Fluorescence was monitored during the PCR every 0.1°C temperature change. PCR mixtures contained 0.5 µM primers for either β-actin sense: 5′-TCC TGT GGC ATC CAC GAA ACT-3′, antisense: 5′-GAA GCA TTT GCG GTG GAC GAT-3′; human ICAM-1, sense: 5′-GGC TGG AGC TGT TTG AGA AC-3′, antisense: 5′-ACT GTG GGG TTC AAC CTC TG-3′ or human eotaxin-3, sense:5′-GGA ACT GCC ACA CGT GGG AGT GAC 3′, antisense: 5′-CTC TGG GAG GAA ACA CCC TCT CC3’. The samples were denatured at 95°C for 10 min, followed by 45 cycles of annealing and extension at 95°C for 12s, 55°C for 5s, and 72°C for 10s. The melting curves were obtained at the end of amplification by cooling the samples to 65°C for 15s, followed by further cooling to 40°C for 30s. Serial 10-fold dilutions were prepared from previously amplified PCR products of β-actin, ICAM-1 and eotaxin-3, which were then used as standards to plot against the unknown samples. Data were quantified with lightcycler analysis software, and values were normalized to the level of β-actin expression for each sample on the same template cDNA.

### Double Immunostaining for TGFβ-1 Positive Cells and Nerves

Some sections from our first study group were also stained for TGFβ-1 in conjunction with S100 (CD n = 4, UC n = 5, Controls n = 2). The tissue sections were de-waxed by immersion in xylene, re-hydrated in alcohol and washed in distilled water. Non-specific peroxidase activity was then blocked by immersing the slides in a 3% hydrogen peroxide solution for 10 minutes. Sections were then unmasked using 0.01% pronase, made up in buffer, pH 7.2, for 10 minutes. After two washes in TBS, the tissue was then incubated with 10% normal horse serum for 10 minutes followed by the application of the TGFβ-1 antibody at a concentration of 1∶40 and incubated overnight at 4°C. After incubation with the TGFβ-1, the tissue was then washed twice in TBS, incubated for 20 minutes with a biotinylated horse secondary antibody diluted in normal horse serum and TBS, washed twice in TBS and incubated for a further 20 minutes with an avidin biotinylated alkaline phosphatase complex. Detection was with the Vector Red substrate working solution made up with Tris-HCL buffer, pH 8.2–8.5. This produced a red coloration within cells staining for TGFß-1. The S100 immunostaining and detection was carried out as described above.

### Double Immunofluorescent Staining for TGFβ-1 and MBP

For this part of the study we examined tissue from both study groups (active responsive IBD, n = 2; quiescent IBD, n = 2; refractory IBD, n = 2; control, n = 1). Formalin-fixed paraffin-embedded tissue was prepared in the same way as described above and the antibody concentrations, unmasking steps and detection systems are outlined in **Table 1**. After incubation overnight with anti-TGFβ-1 antibody, sections were incubated for 20 minutes with a biotinylated universal secondary antibody, washed twice in TBS and incubated in the dark for a further 20 minutes with 1% Fluorescein Avidin D. This produced a green immunofluorescent signal. After two washes in TBS, sections were blocked using the Avidin/Biotin blocking kit according to the manufacturer’s instructions. After a further wash, sections were incubated for 10 minutes with 10% normal horse serum, followed by application of the polyclonal anti-MBP antibody and incubated for 1 hour. The sections were then washed twice in TBS, incubated for 20 minutes with a biotinylated universal secondary antibody, washed twice in TBS and incubated for a further 20 minutes with 1% Texas Red Avidin D. This produced a red immunofluorescent signal. Sections were then washed twice in TBS, incubated in DAPI (200 ng/ml in TBS) for five minutes, allowed to air-dry and then mounted using aqueous mounting medium. All steps were carried out in the dark once the Fluorescein Avidin D had been used on the sections.

### Quantification

We used an established technique for the analysis of the association of eosinophils with nerves [26]. In brief full thickness sections from surgical resection specimens (refractory IBD patients and controls) were examined under low magnification to allow orientation of the section and measurement of surface area. A systematic analysis from lumen to the serosal surface of a two separate 500 µm wide sections of tissue was then made under high power. On average, between 15 and 20 high power fields were examined on full thickness sections. Each high power field was viewed under the microscope and using a digital camera (Nikon camera DXM1200) attached to the microscope, the image was photographed and transferred to a computer program for storage and image analysis, using the Lucia Image Analyser (Laboratory Imaging Limited, Prague, Czechoslovakia). Eosinophils touching, associated with (within 15 µm) nerves and not associated with nerves were counted by two investigators blinded to the nature of the disease.

In the case of the biopsies taken from patients with active and quiescent disease, we used the same technique to quantify the association of eosinophils and nerves. Instead of counting systematically from lumen to serosal surface, one high power field per biopsy with mucosa at the edge of the field was counted and 3 separate biopsies were examined per patient. This technique was employed to ensure as much standardization and reproducibility as possible given the significant variability in the size and relative dimensions of the biopsies.

The association of TGFß-1 positive cells with nerves was similarly counted using 5 random fields within the mucosa and 5 within the smooth muscle layer.

In the case of the immunofluorescent stained sections, we counted 315 eosinophils (stained with anti-MBP) and quantified the proportion of these cells that were also expressing TGFβ-1.

### Statistical Analysis

Comparisons of eosinophils/mm^2^ in contact with nerves were compared between subject groups using ANOVA. Values are expressed as mean +/− SEM.

## Results

### Quantification of Eosinophil Numbers in IBD

**Figure 1:**
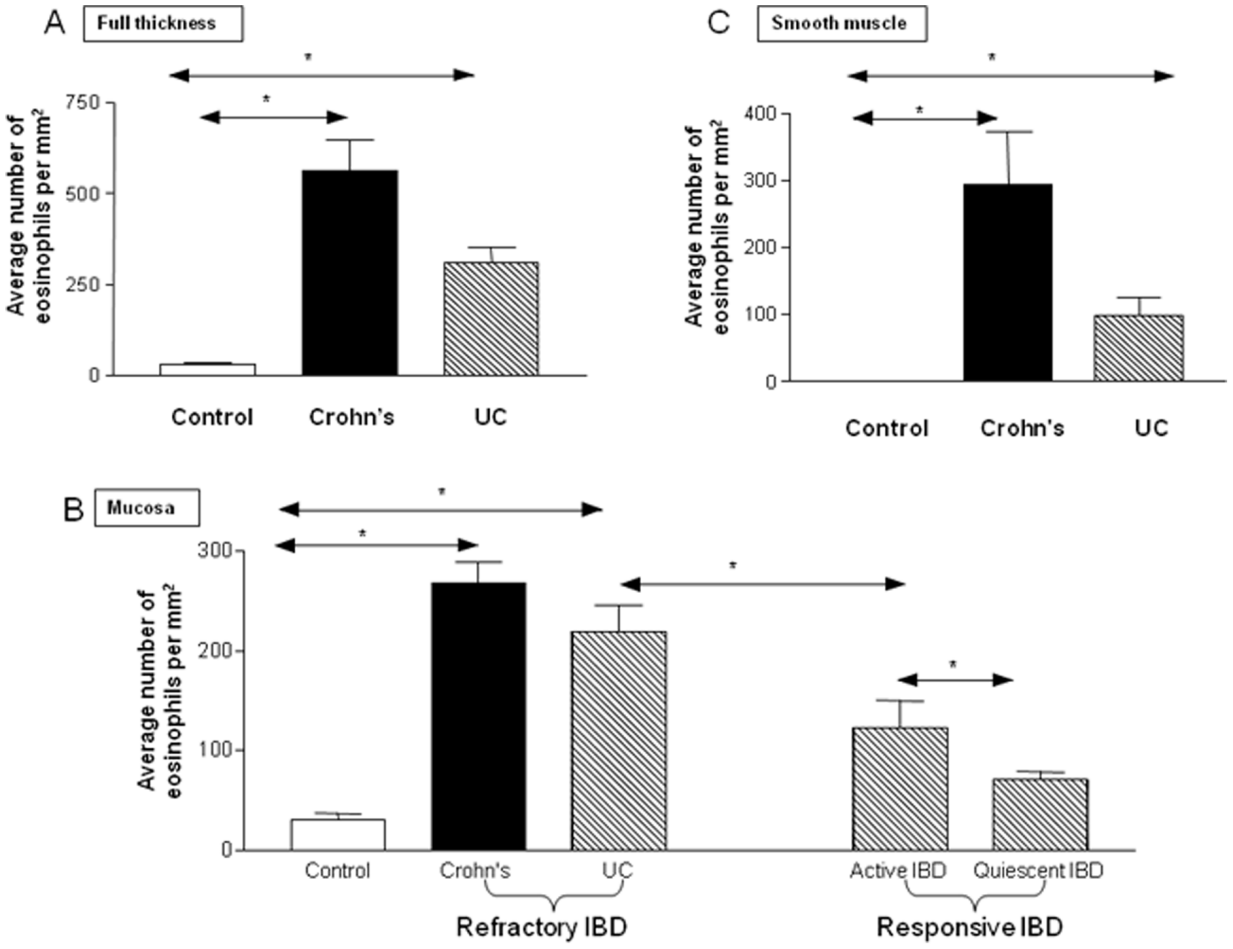
Quantification of Eosinophil localization to bowel wall in patients with IBD. Graph (A) shows the average number of eosinophils per mm^2^ in the entire wall of the colon in patients with refractory IBD compared with controls. Graph (B) shows the average number of eosinophils per mm^2^ within the mucosal layer of patients with refractory IBD and therapeutically responsive IBD compared with controls. It demonstrates that eosinophilic infiltrate correlates with disease activity in IBD. Graph (C) shows the average number of eosinophils per mm^2^ within the smooth muscle layer of patients with refractory IBD compared with controls. The quantification was performed as outlined in the materials and methods section. Control patients n = 8; responsive IBD n = 11; refractory CD n = 8; refractory UC n = 7.The data are expressed as mean ± SEM, *p<0.05.

The sensitivity and specificity of the antibody to MBP was confirmed using nasal polyps as a positive control and omission of the primary antibody as negative control, as reported previously [38]. An anti-S100 antibody was used as a general nerve marker for this part of the study [44]. Neural tissue was used as a positive control and omission of the primary antibody as negative control. Examination of 649 high power fields from 15 subjects with refractory IBD and eight controls showed a significant accumulation of eosinophils throughout the bowel wall of patients with refractory CD (562.2+/−84.7 eosinophils/mm^2^) and UC (309.7+/−43.6 eosinophils/mm^2^) compared to controls (31.5+/−5.7 eosinophils/mm^2^), p<0.05 (**Fig. 1A**). Within the mucosa, eosinophil numbers were increased in refractory CD (268+/−21 eosinophils/mm^2^), UC (219+/−27.1 eosinophils/mm^2^) and therapeutically responsive UC both when symptomatic (122.3+/−28 eosinophils/mm^2^) and when quiescent (71.5+/−7.3 eosinophils/mm^2^). All of these were significantly increased compared to controls, in whom the mean number was 31.5+/−5.7 eosinophils/mm^2^ (**Fig. 1B**). There were significantly more eosinophils in the refractory group compared to the responsive group. Furthermore, in the responsive group, eosinophil numbers fell from 122+/−28 when the disease was clinically active to 71.5+/−7.3 eosinophils per mm^2^ when the disease was quiescent (**Fig. 1B**). Within the smooth muscle layer, there was a significant accumulation of eosinophils in both refractory CD (294+/−78 eosinophils/mm^2^) and UC (98+/−27 eosinophils/mm^2^) compared to controls (0.44+/−0.4 eosinophils/mm^2^), p<0.001 and p<0.05 respectively (**Fig. 1C**). There was no significant difference between the various sub-classifications of CD patients (i.e. those with strictures and active inflammatory disease).

### Eosinophil Association with Nerves

**Figure 2:**
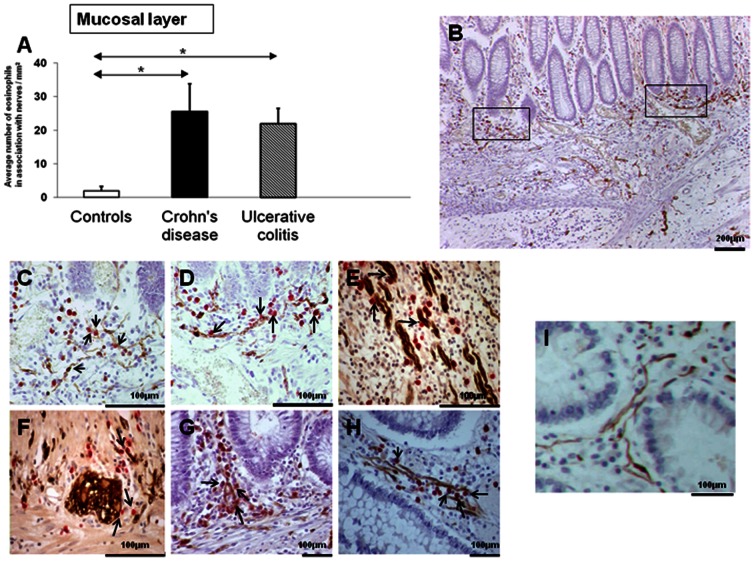
Quantification of Eosinophil localization to bowel wall in patients with IBD.Quantification of eosinophil localization to mucosal nerves in patients with refractory IBD. Eosinophils localize to nerves in the colonic mucosa of patients with refractory IBD. The number of MBP immmunoreactive eosinophils in association with nerves/mm^2^ is shown for controls and subjects with refractory (unresponsive) CD and UC (A). Control patients n = 8; CD n = 8; UC n = 7.The data are expressed as mean ± SEM, *p<0.05. The photomicrograph shown in (B) represents a section taken from a patient with refractory CD. The boxed areas from (B) are shown in higher magnification in (C) and (D). Eosinophils, stained red were detected with anti-MBP antibody, while nerves, stained brown were detected with anti-S100 antibody. There is significant accumulation of eosinophils in these sections compared to the control section shown in photomicrograph (I). Photomicrographs (E) and (F) demonstrate neuroeosinophilic co-localization in a refractory CD section in more detail. Photomicrographs (G) and (H) represent sections from a patient with refractory ulcerative colitis. The arrows in (C-H) demonstrate MBP stained eosinophils in contact or in close association with S100 stained nerves. In all sections note the absence of detectable extra-cellular MBP deposition.

**Figure 3:**
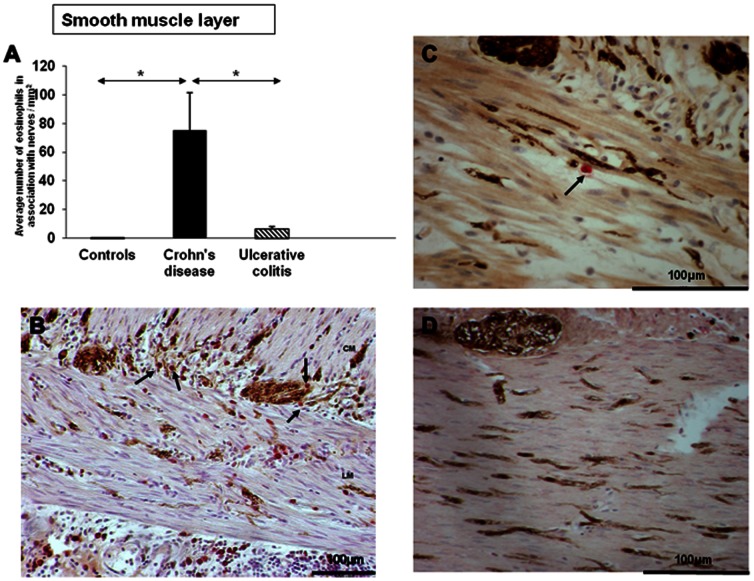
Quantification of Eosinophil localization to bowel wall in patients with IBD.Quantification of eosinophil localization to mucosal nerves in patients with refractory IBD.Eosinophils localize to the muscle layer in IBD and specifically to nerves in CD. The graph (A) shows the total number of eosinophil per mm^2^ localizing to nerves in the muscle layer of patients with refractory IBD. The data are from 8 control patients, 8 patients with CD and 7 with UC. Values are mean ± SEM, * p<0.05. The photomicrograph shown in (B) shows eosinophil localization to nerves in the smooth muscle layer of patients with refractory CD. Photomicrograph (C) demonstrates neuroeosinophilic association in closer detail. Photomicrograph (D) shows a section from a control patient. The arrows in (B,C) demonstrate MBP stained eosinophils in contact or in close association with S100 stained nerves.

There was a significant localization of MBP immunoreactive eosinophils to nerves in the mucosa of patients with refractory IBD (CD 25.6+/−8.3; UC 21.9+/−4.6; compared to controls 2+/−1.3), both p<0.05 (**Fig. 2**). In the muscle layer of patients with CD there was a significant localization of eosinophils to nerves, compared to both UC and controls (CD 75+/−26.4; UC 6.7+/−1.5; Controls 0), p<0.05 and p<0.01 respectively (**Fig. 3**).

### Eosinophil Degranulation in IBD

**Figure 4:**
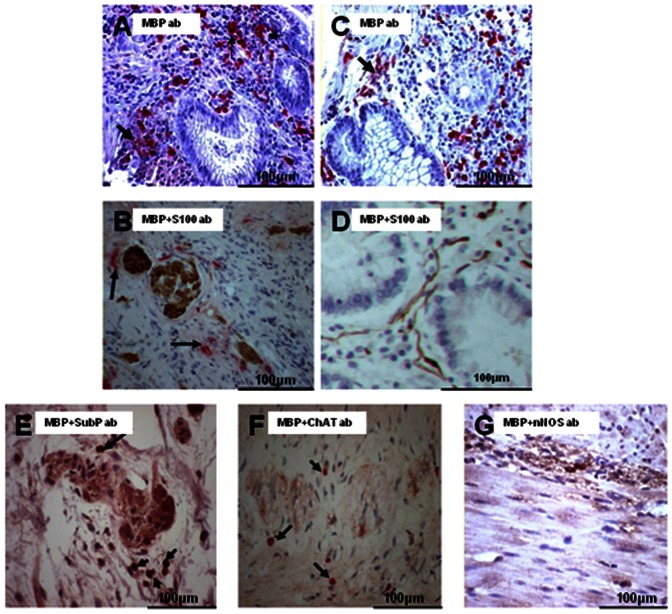
Eosinophil phenotype and nerve subspecies in IBD. There is a considerable eosinophil accumulation and deposition of extra-cellular MBP in patients with therapeutically responsive IBD. Photomicrographs (A and B) are representative sections from patients with clinically active responsive UC and arrowed areas demonstrate extracellular MBP. Photomicrograph (B) is also immune-stained with anti-S100 to demonstrate neural tissue. Photomicrograph (C) is taken from a patient with clinically quiescent therapeutically responsive UC. The arrows in photomicrographs (A-C) illustrate examples extracellular MBP. Note that extracellular MBP deposition is less prominent in the clinically quiescent state. (D) is a representative section from a control patient. Photomicrographs (E) and (F) demonstrate eosinophil co-localization to Substance P and ChAT immunoreactive nerves respectively in patients with IBD. Eosinophils did not co-localize to nNOS immunoreactive nerves (G). The arrows in (E, F) demonstrate MBP stained eosinophils in association with nerve subspecies.

Deposition of extra-cellular MBP was seen in both active and quiescent stages in the clinically responsive group of patients **(** **Fig. 4** **A–C)**. This is more prominent in clinically active disease. In contrast, deposition of extra-cellular MBP was rarely seen in the refractory group (**Fig. 2** **B–H) and (** **Fig. 3** **B,C)**.

### Eosinophils and Nerve Subspecies

Qualitative analysis of eosinophil localization to specific nerve sub-types in IBD showed that eosinophils localized to substance P and ChAT but not nNOS immunoreactive nerves (**Fig. 4E–G**).

### ICAM-1 and Eotaxin-3 Expression by Enteric Ganglia

**Figure 5:**
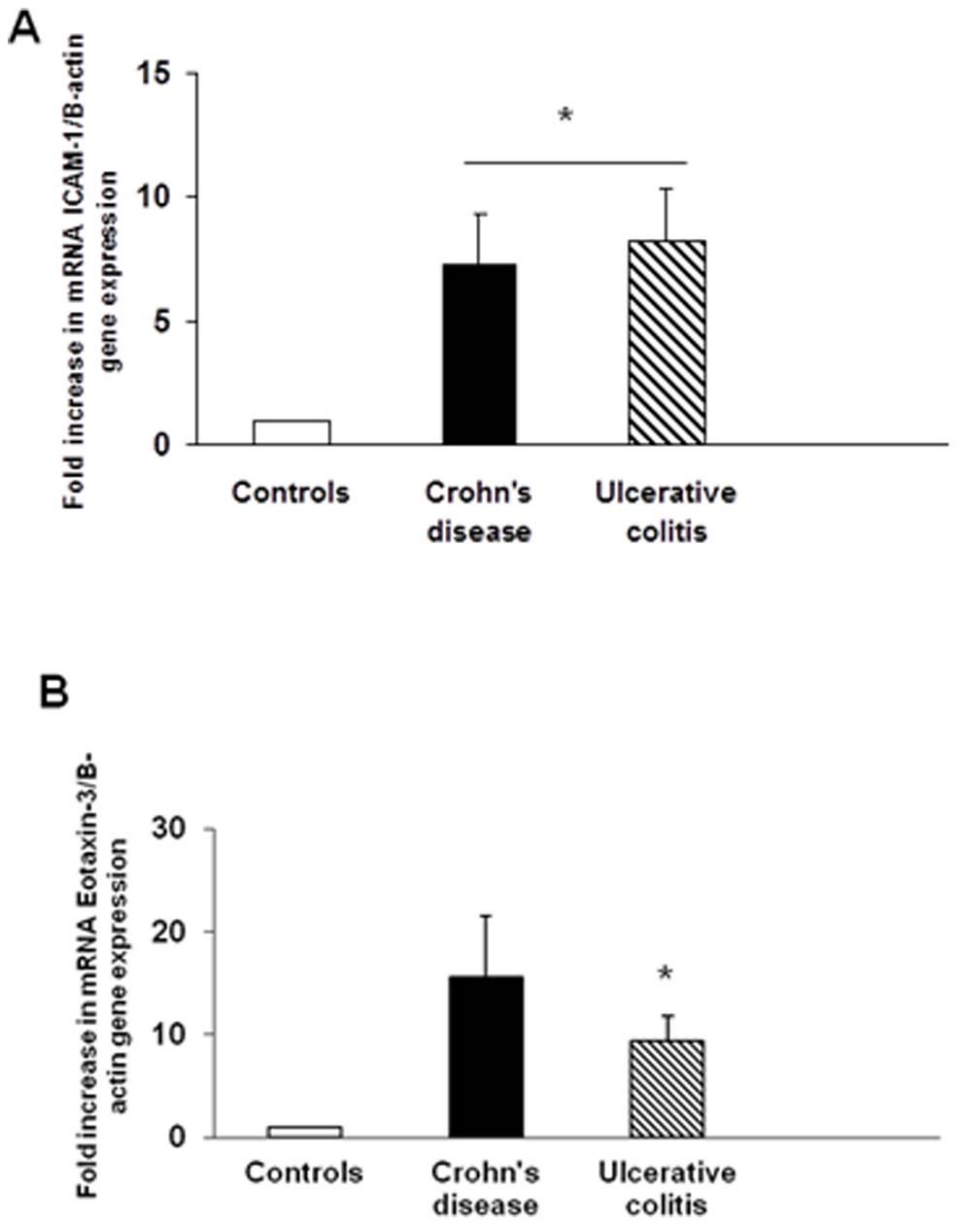
Quantification of Eosinophil localization to bowel wall in patients with IBD.Quantification of eosinophil localization to mucosal nerves in patients with refractory IBD.Eosinophils localize to the muscle layer in IBD and specifically to nerves in CD.Up-regulation of eotaxin-3 and ICAM-1 expression in patients with refractory IBD. In graphs A and B, ICAM-1 and eotaxin-3 expression are shown as the fold increase of gene product normalized for β-actin from the same cDNA sample above control (normal bowel tissue). Controls n = 5; UC n = 4; CD n = 3, *p<0.05.

Paraffin embedded sections of tissue from refractory IBD and control subjects were stained with an anti-S100 antibody to identify neural ganglia. Twenty to forty immunolabelled ganglia were dissected from each section. Real time PCR analysis of the cDNA from these samples showed that mRNA ICAM-1 was increased 7-fold in the neural ganglia of patients with refractory UC, (p = 0.03) and 10-fold in refractory CD, (p = 0.04). Similarly, mRNA eotaxin-3 was increased 9-fold in the nerve ganglia of patients with refractory UC, (p = 0.04) and 15-fold in refractory CD, (p = 0.06). Both genes were standardized against the concentration of the β-actin gene from the same sample (**Fig. 5**).

### TGFβ-1 Expression in Inflammatory Bowel Disease

**Figure 6:**
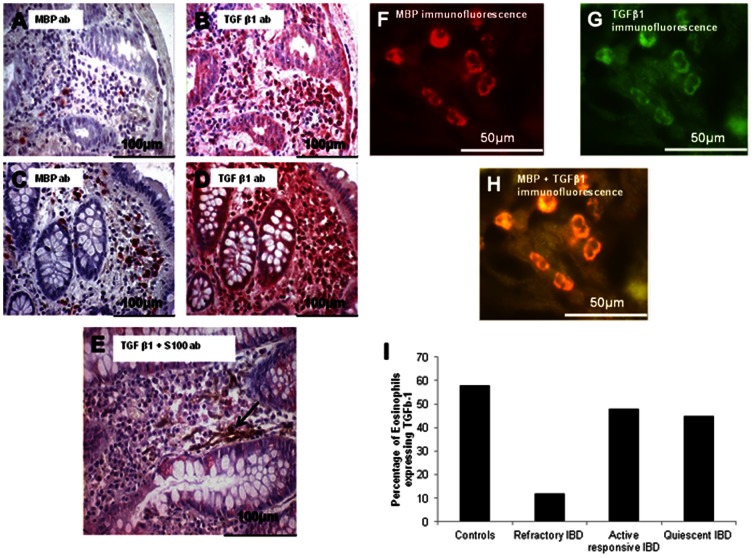
Eosinophilic expression of TGFβ-1. Photomicrographs (A-D) are paired serial sections taken from patients with active (A, B) and quiescent (C, D) UC. (A) and (C) were stained with an anti-MBP antibody while (B) and (D) were stained with an anti-TGFβ-1 antibody. An illustratation of TGFβ-1 immunoreactive eosinophil localization to neural tissue is arrowed in (E). Photomicrographs (F-H) represent a section from a patient with quiescent UC which was double immunofluorescently stained with anti-MBP and anti-TGFβ1. In (F) MBP positively staining cells confirms that these cells are eosinophils. In (G) the same section demonstrates TGFβ1 expression. (H) is a merged image of (F) and (G) confirming that these are TGFβ1 expressing eosinophils. (I) represents the results of the quantitative analysis of eosinophilic TGFβ1 expression. Eosinophilic TGFβ1expression is markedly reduced in patients with refractory IBD (12%) compared to controls (58%) and patients with therapeutically responsive IBD (>40%).

In responsive IBD there were increased numbers of TGFβ-1 immunoreactive cells but there was little difference in the proportion of cells expressing TGFβ-1 between active and quiescent disease (**Fig. 6B,D**). Photomicrographs in **(** **Fig. 6A,C** **)** show paired sections this time stained with anti-MBP demonstrating the relative contribution of eosinophils to overall TGFβ-1 expression in these patients. In refractory disease the overall level of expression of TGFβ-1 was lower than seen in responsive disease, but was still increased compared with controls (CD 8.6+/−4.6; UC 8.3+/−2.2; controls 2.1+/−0.4 cells/mm^2^), (p<0.05). Some TGFβ-1 positive eosinophils were seen localizing to nerves (**Fig. 6E**). Quantitative analysis of TGFβ-1 expression by eosinophils was undertaken on immuno-fluorescently stained sections from a small number of patients with responsive (active and quiescent) and refractory disease. The photomicrographs in **(** **Fig. 6** **F–H)** demonstrate a representative section from a patient with quiescent UC and show how TGFβ-1 expressing eosinophils were clearly identified. TGFβ-1 expression was seen in 12% of eosinophils in refractory IBD while 48% of eosinophils expressed TGFβ-1 in active responsive IBD and 45% in quiescent IBD (**Fig. 6I**). This suggests a failure of TGFβ-1 production in particular by eosinophils is a feature of refractory IBD.

## Discussion

The main finding of this study is that in IBD eosinophils selectively localized to nerves within the mucosa, and in CD this neural inflammation extended to the muscle layer. Eosinophils localized to substance P and ChAT but not nNOS immunoreactive nerves. Neural ganglia expressed the chemoattractant eotaxin-3 and the adhesion molecule ICAM-1, and the levels of expression of these genes were increased in IBD, suggesting a mechanism of eosinophil recruitment to nerve cells. Furthermore, eosinophil numbers and their phenotype reflected disease activity. These data suggest that the localization of eosinophils to nerves may be a mechanism for the symptoms of IBD.

Since we wished to understand the role of eosinophils in IBD, tissue from several different stages of the disease were studied. A patient group with disease refractory to medical treatment requiring surgical resection and a group who responded to standard therapy were studied. In this latter group biopsies were taken when the condition was clinically active and again when the condition was clinically quiescent.

The total number of eosinophils in the mucosa of patients reflected the disease activity, being highest in those with refractory IBD, lower in clinically active therapeutically responsive disease, and, while higher than controls, eosinophil numbers were lowest in those with quiescent disease. Within the smooth muscle layer there was a significant accumulation of eosinophils in both CD and UC. CD is considered to be a transmural inflammatory condition, but it was not expected that a similar accumulation would be seen in UC. Co-localization of eosinophils and nerves in the smooth muscle layer only occurred in patients with CD and it is not clear why co-localization did not occur in those with UC. Even though the difference in eotaxin-3 mRNA levels between the two disease groups was not statistically significant, the reduced level in those patients with UC may be an explanation for this lack of co-localization in the smooth muscle layer in these patients.

In addition to differences in eosinophil numbers across different stages of disease the phenotype of eosinophils, including expression of TGFβ-1 and deposition of extra-cellular major basic protein (MBP), were also different. TGFβ-1 has a potent suppressive effect on Th_1_ effector cells [45], [46] and may be involved in tissue repair through fibrosis. Studies have also suggested a role for TGFβ-1 in airway remodelling in asthma in animal models [25]. This is the basis of the rationale of studying TGFβ-1 expression in the different clinical stages of IBD. The relative paucity of expression, in particular eosinophilic expression of TGFβ-1 in refractory IBD may be a factor in the pathophysiology of this stage of the disease. Further studies will be needed to examine this observation. The reasons for differences in the extent of deposition of extra-cellular MBP are more difficult to explain. The stimuli for granular protein release are uncertain, but the different inflammatory environment of refractory and therapeutically responsive IBD may have different effects on eosinophils [47]–[49]. Alternatively, since eosinophil proteins are antibacterial and since in IBD the mucosal barrier is breached, eosinophil granular proteins may be exerting a beneficial effect. Failure to release these proteins may predispose the host to develop refractory disease. Further studies will be required to clarify these issues.

In all groups, eosinophils specifically localized to the nerves in the mucosal layer. In addition, in patients with refractory CD, eosinophils also localized to the nerves of the smooth muscle layer. Although, eosinophil localization to nerves has been previously reported in IBD [50], to our knowledge this has not been quantified and related to disease activity. This quantification was performed using an antibody to the general neural marker S100, but in order to investigate which nerves were involved we also immunostained for the common nerve sub-types including substance P, ChAT and nNOS. These studies indicated that eosinophils localized to all sub-types except nNOS nerves. Formal quantification of this observation was not undertaken and the mechanism of this observation is uncertain. In-vitro co-culture studies have shown that eosinophils can induce release of substance P [36]. Thus, the in-vivo finding of eosinophil localization to excitatory nerves such as substance P may be of some clinical relevance since in the gastrointestinal tract substance P is involved in motility, fluid and electrolyte secretion and increased local blood flow [51]–[53]. In addition, increased expression of substance P has been described in inflammatory bowel disease [54]. A possible consequence of the interactions between the eosinophils and ChAT containing nerves is loss of function of inhibitory M_2_ muscarinic receptors on postganglionic nerves. Neuronal M_2_ muscarinic receptors control the release of acetylcholine from the vagus. In-vivo and in-vitro studies have shown that eosinophil MBP is an allosteric antagonist at these receptors [30]. Thus, eosinophils may be responsible for loss of function of these receptors leading to increased smooth muscle contraction and gut motility. Testing this hypothesis will require further investigation, in particular in CD, where eosinophil MBP is seen in association with nerves in the smooth muscle. Eosinophils did not localize to nNOS containing nerves in this study. The reason for this is unclear. Studies have demonstrated increased NOS activity in tissue samples from patients with CD and UC [55], [56]. Some studies have shown that inhibition of NO attenuates intestinal inflammation [57]. NO has also been shown to regulate inflammatory cell apoptosis in a concentration dependent manner [58]. A recent study has also demonstrated that neuro-eosinophilic interactions in patients in UC are bidirectional with transmission of neurological cholinergic signals to muscarinic receptors on corticotropin-releasing factor (CRF) positive eosinophils with subsequent CRF mediated mast cell degranulation and increased mucosal permeability [59].

In this study, apart from determining an interaction and localization between eosinophils and nerves we defined a possible mechanism to explain this localization. Using the methods of laser capture microscopy and quantitative PCR we demonstrated that the mRNA of ICAM-1 and eotaxin-3 was present in the ganglia of all subjects. There was a significant increase in the mRNA levels of these in patients with IBD, compared with controls. ICAM-1 is a ligand for eosinophil CD11/18 integrins, and these have been shown to be up-regulated in patients with active IBD [60], [61]. Studies using an antibody to ICAM-1 showed a beneficial effect in patients with IBD [62], inhibition of eosinophil recruitment to nerves may be one explanation of how this agent exerted this beneficial effect in IBD.

The eotaxins –1 (CCL-11),-2 (CCL-24) and -3 (CCL-26) are selective eosinophil chemoattractants, acting on CCR3 chemokine receptors [63], [64]. Eotaxin-3 is expressed by endothelial cells, fibroblasts and has recently been reported to be expressed by airway nerves [65]. In the airways, of the three eotaxins, eotaxin-3 has been shown to be the most dynamically regulated. In this study we have shown that eotaxin-3 is expressed by enteric nerves and in patients with refractory IBD the level of expression is increased. The finding of an eosinophil chemoattractant and adhesion molecule suggests a mechanism of recruitment of eosinophils to the enteric nerves. While we have demonstated upregulation of ICAM-1 and eotaxin-3 and demonstrated eosinophil localization to nerves in IBD, we cannot conclude a direct cause and effect between the two phenomena based on this work in the absence of inhibitory mechanistic studies. Future studies could investigate the effect of CCR-3 inhibition or monoclonal humanized antibody therapy to CD 11/18, ICAM-1 or VLA-4 (with Natalizumab) on eosinophil recruitment to nerves in IBD. Notably, Natalizumab has established therapeutic benefit in CD. It is important to note that, in addition to enteric nerves, autonomic and sensory nerves also project into the muscular and mucosal layers of the bowel and may also contribute to eotaxin and ICAM-1 regulation. They play a key role in regulation of bowel function including transmission of signals from central nervous system to enteric nerves and production of vasoactive substance P. Autonomic sympathetic neurons may also contribute to M2 receptor regulation by serving as another source of acetylcholine. There is emerging evidence that the autonomic nervous system, in fact, plays an important role in the development of IBD [66]. These factors add considerable complexity to interpretation of neuroeosinophilic interactions in IBD.

Reduction in eosinophil recruitment to the airways using an antibody to eosinophil growth factor IL-5 has been shown to lead to a reduction in sub-epithelial fibrosis [67], suggesting that eosinophils play an important role in inflammation associated remodelling. In the gastrointestinal tract local IL-5 mediated eosinophilia has been shown to be central to oesophageal remodelling in eosinophilic oesophagitis [68]. A recent animal study has demonstrated that attenuation of ileal eosinophilia via CCR-3 inhibition leads to a reduction in fibronectin expression and substantial reduction in the histological markers of remodelling [69].

Since eosinophils localize to nerves and since the present and prior studies have shown nerve damage in IBD [50], this suggests that inhibition of this recruitment and localization may have important beneficial effects in IBD.

In summary, eosinophils localized to nerves and ganglia in the mucosa of patients with IBD. Increased eotaxin-3 and ICAM-1 expression by the enteric nerves was seen in patients with refractory IBD, suggesting a possible mechanism for the association of eosinophils and nerves. Since eosinophil numbers and phenotype in the mucosa correlated with the activity of the disease these data suggest that eosinophils may play an important role in the pathogenesis of IBD through activation of these nerves.

## Supporting Information

Table S1 (DOCX)
