# Supplementary material for: Activated Eosinophils in Association with Enteric Nerves in Inflammatory Bowel Disease
Source: PLoS One. 2013 May 22;8(5):e64216. doi: 10.1371/journal.pone.0064216 (PMC3661526; doi:10.1371/journal.pone.0064216)
Supplement: Table S1 — (DOCX) [file pone.0064216.s001.docx]

**Table 3:** This table outlines the clinical characteristics of the patients with active and quiescent disease used in this study.

**Table 3: Characteristics of patients with responsive IBD**

| **PATIENT** | **DISEASE ACTIVITY** | **HISTORY & INVESTIGATIONS** | **FEATURES** |
| --- | --- | --- | --- |
| 1 | Active | Clinical symptoms | Abdominal pain. Loose bloody diarrhoea |
|  |  | Endoscopy | Congested erythematous mucosa |
|  |  | Histology | Severe active IBD |
|  |  | Medications | None |
|  | Quiescent | Clinical symptoms | Asymptomatic |
|  |  | Endoscopy | Normal |
|  |  | Histology | Quiescent IBD |
|  |  | Medications | Mesalazine |
| 2 | Active | Clinical symptoms | Diarrhoea, mucus per rectum |
|  |  | Endoscopy | Inflamed ulcerated colon |
|  |  | Histology | Active IBD |
|  |  | Medications | Mesalazine |
|  | Quiescent | Clinical symptoms | Asymptomatic |
|  |  | Endoscopy | Normal |
|  |  | Histology | Normal |
|  |  | Medications | Mesalazine |
| 3 | Active | Clinical symptoms | Loose bloody diarrhoea |
|  |  | Endoscopy | Moderately active colitis |
|  |  | Histology | Active colitis with chronicity |
|  |  | Medications | Salazopyrine |
|  | Quiescent | Clinical symptoms | Asymptomatic |
|  |  | Endoscopy | Normal, but mild caecitis |
|  |  | Histology | No active colitis |
|  |  | Medications | Salazopyrine |
| 4 | Active | Clinical symptoms | Loose bloody diarrhoea |
|  |  | Endoscopy | Proctitis with pseudopolyps |
|  |  | Histology | Severe acute IBD |
|  |  | Medications | Salazopyrine and oral steroids |
|  | Quiescent | Clinical symptoms | Asymptomatic |
|  |  | Endoscopy | Normal apart from some pseudopolyps |
|  |  | Histology | Normal |
|  |  | Medications | Mesalazine |
| 5 | Active | Clinical symptoms | Loose bloody diarrhoea |
|  |  | Endoscopy | Moderate to severe colitis |
|  |  | Histology | Active IBD |
|  |  | Medications | Mesalazine |
|  | Quiescent | Clinical symptoms | Asymptomatic |
|  |  | Endoscopy | Normal |
|  |  | Histology | Normal |
|  |  | Medications | Mesalazine |
| 6 | Active | Clinical symptoms | Blood per rectum |
|  |  | Endoscopy | Inflamed rectal mucosa |
|  |  | Histology | Moderately active IBD |
|  |  | Medications | Steroids – oral and rectal |
|  | Quiescent | Clinical symptoms | Asymptomatic |
|  |  | Endoscopy | Normal |
|  |  | Histology | No active IBD |
|  |  | Medications | Olsalazine |
| 7 | Active | Clinical symptoms | Mucus, blood and pus PR |
|  |  | Endoscopy | Moderately active UC |
|  |  | Histology | Moderately active UC |
|  |  | Medications | Salazopyrine, Azathioprine, oral steroids |
|  | Quiescent | Clinical symptoms | Asymptomatic |
|  |  | Endoscopy | Quiescent UC |
|  |  | Histology | Normal |
|  |  | Medications | Salazopyrine, Azathioprine |
| 8 | Active | Clinical symptoms | Loose bloody diarrhoea, mucus pr |
|  |  | Endoscopy | Moderate to severe distal colitis |
|  |  | Histology | Severely active IBD |
|  |  | Medications | None |
|  | Quiescent | Clinical symptoms | Occasional blood PR |
|  |  | Endoscopy | Normal |
|  |  | Histology | Unremarkable |
|  |  | Medications | None |
| 9 | Active | Clinical symptoms | Intermittent abdominal with blood and mucus PR |
|  |  | Endoscopy | Left-sided active colitis |
|  |  | Histology | Severely active IBD |
|  |  | Medications | Salazopyrine |
|  | Quiescent | Clinical symptoms | Asymptomatic |
|  |  | Endoscopy | No active colitis |
|  |  | Histology | Quiescent IBD |
|  |  | Medications | Salazopyrine |
| 10 | Active | Clinical symptoms | Diarrhoea |
|  |  | Endoscopy | Erythema, contact bleeding |
|  |  | Histology | Moderate to severe active colitis |
|  |  | Medications | None |
|  | Quiescent | Clinical symptoms | Asymptomatic |
|  |  | Endoscopy | Mild erythema in rectum |
|  |  | Histology | Normal |
|  |  | Medications | Salazopyrine |
| 11 | Active | Clinical symptoms | Bloody diarrhoea with abdominal pain |
|  |  | Endoscopy | Congested granular mucosa |
|  |  | Histology | Active IBD |
|  |  | Medications | Mesalazine, Steroids IV and PR |
|  | Quiescent | Clinical symptoms | Semi-formed bowel motions with normal frequency |
|  |  | Endoscopy | Mild rectal erythema |
|  |  | Histology | Normal |
|  |  | Medications | Steroids PO and PR |
